# Highly Anomalous Energetics of Protein Cold Denaturation Linked to Folding-Unfolding Kinetics

**DOI:** 10.1371/journal.pone.0023050

**Published:** 2011-07-29

**Authors:** M. Luisa Romero-Romero, Alvaro Inglés-Prieto, Beatriz Ibarra-Molero, Jose M. Sanchez-Ruiz

**Affiliations:** Departamento de Quimica Fisica, Facultad de Ciencias, Universidad de Granada, Granada, Spain; University of South Florida College of Medicine, United States of America

## Abstract

Despite several careful experimental analyses, it is not yet clear whether protein cold-denaturation is just a “mirror image” of heat denaturation or whether it shows unique structural and energetic features. Here we report that, for a well-characterized small protein, heat denaturation and cold denaturation show dramatically different experimental energetic patterns. Specifically, while heat denaturation is endothermic, the cold transition (studied in the folding direction) occurs with negligible heat effect, in a manner seemingly akin to a gradual, second-order-like transition. We show that this highly anomalous energetics is actually an apparent effect associated to a large folding/unfolding free energy barrier and that it ultimately reflects kinetic stability, a naturally-selected trait in many protein systems. Kinetics thus emerges as an important factor linked to differential features of cold denaturation. We speculate that kinetic stabilization against cold denaturation may play a role in cold adaptation of psychrophilic organisms. Furthermore, we suggest that folding-unfolding kinetics should be taken into account when analyzing in vitro cold-denaturation experiments, in particular those carried out in the absence of destabilizing conditions.

## Introduction

The existence of cold denaturation is a straightforward prediction of a widely-accepted phenomenological view of protein folding thermodynamics [1]. The simplest rendering of the prediction is as follows. The enthalpy change for protein unfolding (ΔH) is temperature-dependent (as given by the positive unfolding heat capacity change and the Kirchoff equation) and equals zero at the so-called enthalpy-inversion temperature (T_H_). At temperatures above T_H_, ΔH is positive, the unfolding process is endothermic and, hence, it is driven by a temperature increase (heat denaturation). Below T_H_, ΔH is negative, the unfolding process is exothermic and it is driven by a temperature decrease (cold denaturation).

Several facts make cold denaturation an important subject of study. A large number of organisms (in particular, microorganisms in the oceans) are psychrophiles [2] and cold-adaptation likely involves decreased cold-denaturation propensity in many of their proteins. [It is interesting in this context that recent findings of elevated ubiquitin-conjugated protein levels in Antarctic fish have been interpreted as evidence of cold denaturation in vivo [3]]. Also, unfolded and partially-unfolded states may be highly susceptible to irreversible alterations (misfolding and aggregation, for instance) and, hence, cold denaturation may compromise the shelf-life of protein pharmaceuticals upon low-temperature storage. [It is interesting in this context that evidence for cold-denaturation of monoclonal antibodies has been recently reported [4]].

Unfortunately, cold denaturation is difficult to study in vitro as the equilibrium cold-denaturation temperature is commonly below the freezing point of water. With a few relevant exceptions [5], [6], experimental cold denaturation in vitro is achieved with the help of destabilizing solvent conditions, high pressure, mutations or even core fluorination [7]–[13]. Furthermore, despite several careful experimental analyses, a number of fundamental issues remain unsolved and a consensus about the molecular mechanism of cold denaturation has yet to emerge [14]. In particular, it is not clear whether cold-denaturation is just a “mirror image” of heat denaturation or whether it shows unique structural and energetic features. In this context, the residual structure in the cold-denatured state and the cooperativity of the cold denaturation process have been frequently addressed in the literature [15]–[27].

Here we report that, for a well-characterized small protein, heat-denaturation and cold-denaturation show dramatically different experimental energetic patterns. Specifically, while heat denaturation is endothermic, the cold transition (studied in the folding direction) occurs with negligible heat effect (ΔH∼0), in (apparent) serious conflict with the widely-accepted view of protein thermodynamics summarized in the opening paragraph. We show that this highly anomalous behavior does not primarily arise from residual-structure or decreased-cooperativity effects; rather, it is an apparent effect that mainly reflects differential kinetic features of cold denaturation that have received little attention in previous studies.

## Results and Discussion

**Figure 1:**
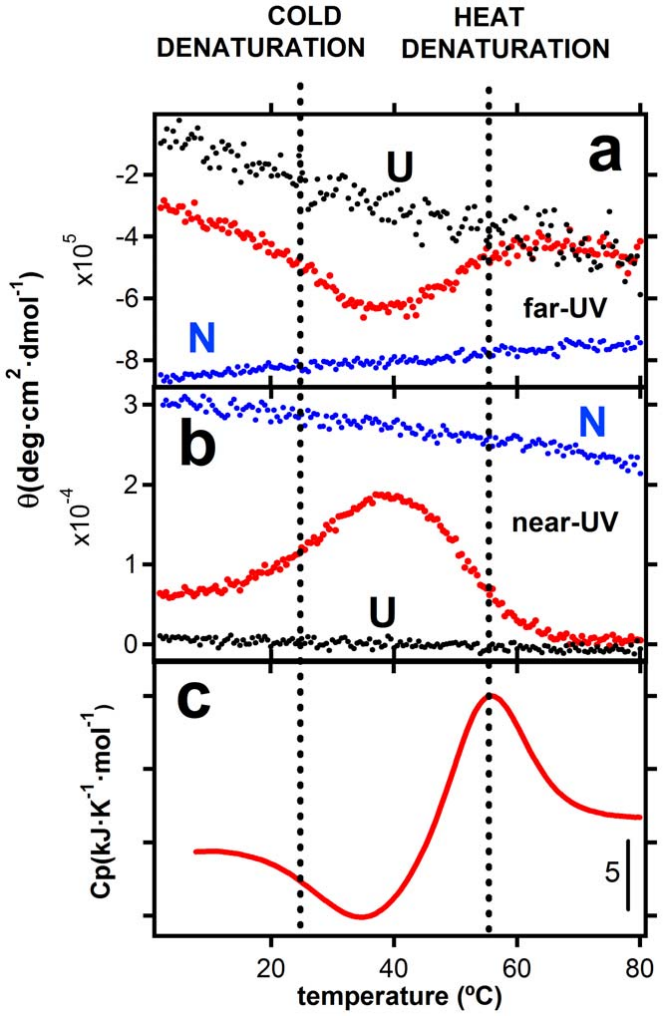
Heat and cold denaturation of thioredoxin at pH 7 in the presence of 2M guanidine, as followed by, a) far-UV circular dichroism (222 nm), b) near-UV circular dichroism (280 nm) and, c) differential scanning calorimetry. The three experiments (far-UV, near-UV and DSC) were carried out with the same solution and following exactly the same heating protocol (see text and “Materials and Methods” for details). The native-state (N) and unfolded-state (U) profiles in panels a and b were obtained in the absence of guanidine and in the presence of 6M guanidine, respectively.

Figures 1a and 1b show the results of far-UV and near-UV circular dichroism experiments in which E. coli thioredoxin solutions were heated at a constant temperature scanning-rate of 1.5 degrees/min. [Thioredoxin solutions were kept at 2°C for 3 hours prior to the start of the temperature scanning experiment; we checked, nevertheless, that a several-days incubation at 2°C led essentially to the same results]. Protein ellipticity is an average over protein states and, consequently, folding-unfolding transitions are revealed by sigmoidal-like changes in profiles of ellipticity vs. temperature. The profiles for solutions including 2M guanidine reveal two well-defined sigmoidal-like changes obviously corresponding to the cold and heat denaturation processes. A differential scanning calorimetry (DSC) profile for the same thioredoxin solution and using the same temperature protocol is shown in Figure 1c. DSC measures the heat capacity of a protein solution, which is the temperature-derivative of the average enthalpy. Hence, a folding-unfolding transition is usually revealed by a heat capacity “peak” (the derivative of a sigmoidal-like profile), the area under which equals the total enthalpy change for the transition. Then, DSC thermograms for heat-and-cold denaturation typically show two peaks, often loosely resembling the double hump of a Bactrian camel. The experimental calorimetric profile in Figure 1c is, therefore, most unusual, as a peak is observed only for heat denaturation. Cold denaturation in this profile is actually signaled by a gradual increase in heat capacity upon temperature decrease (roughly from the heat capacity level expected for the native protein to the level expected for the unfolded protein). That is, contrary to heat denaturation, cold denaturation appears to occur with negligible heat effect (close to zero transition enthalpy change).

The two following scenarios may provide a plausible explanation for the astonishing experimental result shown in figure 1: Scenario A) Cold denaturation is a gradual, non-cooperative process, which could be loosely described as a “second-order-like” transition. Actually, this type of interpretation has been previously discussed in connection with the gradual heat-capacity profiles for the denaturation of a compact intermediate state of apomyoglobin and also for the denaturation of FSD-1ss, a designed mini protein studied as a model of primordial folding [28], [29]. Scenario B) The free-energy barrier for folding-unfolding is large and, at low temperature, the system is kinetically trapped in the initial distribution of states. Upon temperature increase, the rate of folding/unfolding increases and becomes significant at temperatures close to the enthalpy inversion temperature. Consequently the cold transition occurs in an out-of-equilibrium, kinetically-controlled manner with little heat effect.

**Figure 2:**
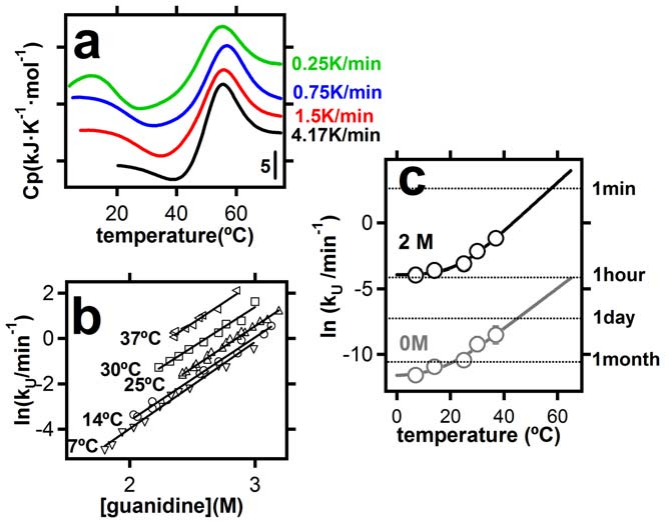
Cold denaturation and folding-unfolding kinetics. a) Scan-rate effect on the DSC profiles for thioredoxin heat and cold denaturation at pH 7 and in the presence of 2M guanidine. All the scanning experiments started at 2°C. The differences observed in the lowest temperature are related to the instrument equilibration time (see Materials and Methods for details). b) First-order rate constants for thioredoxin unfolding as a function guanidine concentration for the temperatures shown. c) Temperature-dependence of the rate constants for thioredoxin unfolding at 2M guanidine and in the absence of guanidine(extrapolated in both cases from the linear dependencies shown in panel b). Representative time-scales are shown alongside the right y-axis.

To decide between the two explanations proposed above, we have characterized the scanning-rate effect on the DSC transitions, a clear proxy of kinetic control [30]. The heat-denaturation transition is essentially independent of scanning-rate within the range 0.25–4.17 degrees/min, while a large scanning-rate effect is found for the cold-denaturation transition (Figure 2a), indicating a strong kinetic effect in the latter.

Scenario B is further supported by the rates of unfolding determined from the time-dependence of the protein fluorescence at given, constant temperatures (Figure 2b and 2c). The first-order rate constant for thioredoxin unfolding (k_U_) in 2M guanidine increases with temperature in a manner that is consistent with slow rates at cold-denaturation temperatures and fast rates at heat-denaturation temperatures (see Materials and Methods for a more detailed discussion on the temperature dependence of the unfolding rate constants). Furthermore, the availability of the temperature-dependence of k_U_ allows us to carry out a realistic simulation of a temperature-scanning experiment under scenario B. The simulation procedure we have used is essentially that described by Plaza del Pino et al [31]. Very briefly, a temperature-scanning experiment can be seen as a series of very small, equally-spaced temperature jumps and the relaxation within each jump can be computed from the folding/unfolding kinetics. Then, the relaxation kinetics, together with the folding/unfolding energetics allows denaturation profiles to be calculated (see Materials and Methods for details).

**Figure 3:**
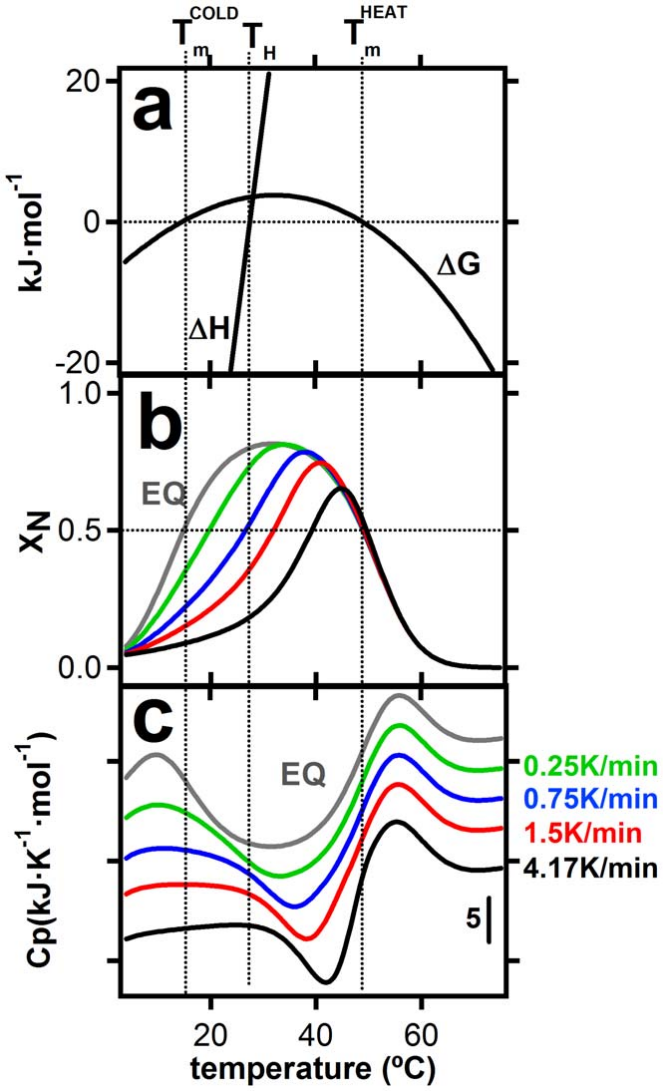
Theoretical simulation of temperature-scanning profiles for protein heat-cold denaturation including slow folding-unfolding kinetics. a) Folding-unfolding energetics used in the simulation (see Materials and Methods for details). Profiles of unfolding free energy and unfolding enthalpy versus temperature are shown. b) Plots of fraction of native protein versus temperature for several values of the temperature scanning-rate. c) Simulated scanning calorimetry profiles at the indicated scan rates. The equilibrium profile (formally corresponding to the limit of zero scan rate) is labeled EQ. Vertical dashed lines crossing the three panels indicate the values of the equilibrium heat- and cold-denaturation temperatures at the enthalpy inversion temperature.

Simulated profiles of fraction of native protein and heat capacity versus temperature thus obtained are shown in Figure 3. They do reproduce the relevant features of the experimental results summarized in figure 1, including in particular a) the strong scan-rate dependence of the cold-denaturation transition, b) the appearance of a cold-denaturation “peak” at the lower scan-rates (i.e., when equilibrium is approached) and c), the shift towards lower temperatures of the heat capacity minimum between the heat and cold denaturation transitions which is observed upon scan-rate decrease.

It is important to note that the simulations shown in figure 3 are based upon reasonable estimates of the energetic parameters of thioredoxin unfolding (see Figure 3a and Materials and Methods for details), together with the temperature dependence of the unfolding rate constant based on experiment (figure 2c). Therefore, the general agreement found does support that the anomalous features of the cold denaturation of thioredoxin are mainly associated to kinetics (although, of course, contributions from other factors, such as residual structure in the unfolded state and decreased cooperativity, cannot be ruled out). Clearly, in a laboratory time scale on the order of minutes-to-hours (corresponding to heating rates of few degrees per minute in a temperature-scanning experiment) kinetic trapping prevents thioredoxin cold transition from taking place at the equilibrium cold-denaturation temperature; rather the process occurs at about the enthalpy-inversion temperature with little heat effect. As elaborated below in some detail, we expect this kinetic effect on cold-denaturation to be a common feature of many proteins that, as is the case with thioredoxin [32]–[34], have been naturally selected to have significant kinetic stability.

Proteins often work in vivo under conditions that favor the occurrence of irreversible alteration processes. For instance, proteolysis, aggregation and incorrect interactions with other macromolecular components may potentially occur in crowded intracellular or harsh extracellular environments. As a result, a protein might not remain in the native, biological functional state in a physiological-relevant time scale, even if the protein is stable in the usual thermodynamic sense (i.e., even if the free energy of the native state is lower than that of the unfolded state under physiological conditions). It is emerging from a number of different experimental studies [35]–[41] that, to avoid this situation, many proteins have been naturally selected to have a high free-energy barrier for unfolding under physiological conditions. Such high barrier “separates” the biologically functional state from inactive states and unfolded (and partially unfolded) states (i.e., the states prone to undergo irreversible alteration processes) and confers kinetic stability to the protein. Certainly, the high free-energy barrier associated to kinetic stability will lead to slow folding-unfolding kinetics at physiological temperatures, although, because of the expected increase of rate with temperature, slow kinetics will not be necessarily observed at the higher heat-denaturation temperatures. Thansthyretin, a protein involved in several misfolding diseases, provides a particularly clear illustration of the point. In vitro heat denaturation of wild type transthyretin occurs at about 100°C and can be adequately described in terms of a two-state mechanism involving equilibrium between the native tetrameric protein and the monomeric unfolded state [42]. Despite of this, recent successful approaches to treat transthyretin amyloidoses (obviously at temperatures much lower than 100°C) use kinetic stabilizers, i.e., substances that have the capability of increasing the unfolding free-energy barrier, thus enhancing transtyretin kinetic stability [43], [44].

It is also worth noting that a strong dissimilarity between the time-scales for the heat- and cold-denaturation processes is linked to a strong temperature dependence of the folding/unfolding rate constants and implies, therefore, high values for the corresponding activation energies. Activation energy values on the order of several hundreds of kJ/mol have been reported for the unfolding of many proteins and attributed in part to the asynchrony between water penetration and the break-up of internal interactions [45]–[47]. Indeed, such solvation/desolvation barriers have been shown to determine several relevant features of protein folding [48] and to be important for protein kinetic stability [41], [45].

We conclude from all the above experiments, analyses and considerations, that the pattern of close-to-equilibrium heat denaturation (in the absence of irreversible alterations, such as aggregation) and kinetically-controlled cold-denaturation may actually be a quite common one. We may ask then why huge kinetic effects do not appear to have been noticed in many previous studies on cold denaturation. The answer is likely related to the fact that cold denaturation is often achieved in vitro with the help of destabilizing conditions that may increase, not only the equilibrium cold denaturation temperature, but also the rate of unfolding. Actually, the attenuation of the kinetic effect is clearly seen in our experimental data, as the presence of guanidine leads to higher rates of thioredoxin unfolding (compare in figure 2c the rates at 2M guanidine with the rates extrapolated at zero denaturant concentration). However, the rates at 2M are still low and the kinetic effect in thioredoxin is not fully eliminated by the destabilizing agent. That is, the kinetic effect we observe on thioredoxin cold-denaturation is not caused by the chemical denaturant but, rather, it occurs despite the presence of a chemical denaturant. The high kinetic stability of thioredoxin [32]–[34], together with the comparatively low guanidine concentration used, may have contributed to this result. It may be reasonable expected that, in many other cases, the destabilizing conditions used to bring cold-denaturation to an experimentally convenient temperature range have efficiently masked the kinetic effect in a laboratory time-scale.

Overall, kinetics emerges as an important factor linked to differential features of protein cold denaturation. Kinetics should be taken into account when analyzing in vitro cold-denaturation experiments, in particular in the absence of destabilizing conditions (using, for instance, water supercooling) or when designing conditions for the low-temperature storage of protein pharmaceuticals. Furthermore, we speculate that kinetic stabilization against cold-denaturation may play an important role in cold-adaptation of psychrophilic organisms.

## Materials and Methods

### Protein solutions

Wild type E. Coli thioredoxin was expressed and purified as previously described [32]. Stock protein solutions in aqueous buffer (50 mM Hepes pH 7) were prepared by exhaustive dialysis against the buffer. Stock solutions of 7M guanidinium chloride in Hepes buffer were prepared by mixing two guanidine solutions in the acidic and basic forms of the buffer used, so that the desired pH-meter reading was obtained. Thioredoxin solutions at 2M guanidine chloride were prepared by mixing appropriate volumes of stock solutions of the protein and 7 M guanidine chloride. Guanidinium chloride concentrations were determined from refraction index measurements using an Abbe 320 refractometer.

### Diferencial scanning calorimetry (DSC)

DSC experiments were carried out in a capillary VP-DSC microcalorimeter (Microcal, Northhampon, USA) with a 0.133 mL cell volumen. Experiments at different scan rates (0.25, 0.75, 1.5 and 4.2 K/min) were performed. In all cases, the protein solution was kept inside the calorimetric cell for 180 min at 2°C before the start of the temperature up-scanning experiments. However, we checked that several-days incubation at 2°C leads to essentially the same results. Note that, although all scanning experiments started at 2°C, the lowest experimental data point depends on the scan rate used (see Figure 2a). This is due to the fact that, during the instrumental equilibration period (about 4 min after the start of the temperature up-scanning), heat capacity values cannot be recorded. The “lost” temperature interval is given by the value of the equilibration period times the scan rate used and, obviously increases with scan rate.

Reheating runs were performed in all cases and demonstrated high operational reversibility of the heat-capacity denaturation profile. The DSC thermograms shown in Figures 1a and 2a are derived from experiments performed at a protein concentration of 4.6 mg/mL, which guarantees an excellent signal-to-noise ratio and minimizes baseline uncertainty effects commonly associated with calorimetric work with water-cosolvent mixtures. Nevertheless, we did carry out additional DSC experiments at different protein concentrations and found no significant protein concentration effect on the calorimetric profiles within the 0.8–4.6 mg/mL range, thus ruling out the possibility of distortions caused by protein oligomerization.

### Circular dichroism (CD) spectroscopy

CD experiments were carried out using a Jasco (Tokyo, Japan) J-715 spectropolarimeter equipped with a PTC-348WI. The change in CD signal within the temperature range of 2–98°C was monitored following a protocol identical to that used in the DSC experiments, i.e., keeping the protein solution in the measurement cell at 2°C for 180 min prior to the start of the upwards temperature-scan. A signal averaging time of 4 s and a bandwidth of 1 nm were used in all cases. For far-UV CD experiments (222 nm), a protein concentration of ∼0.5 mg/mL and 1 mm pathlength cell were used. For near-UV CD experiments (280 nm) a protein concentration of ∼0.9 mg/mL and 0.5 mm pathlength cell were used. In all cases, blanks in the absence of protein were recorded immediately prior to the sample measurements and subtracted prior to the mean molar ellipticity [θ] calculation.

### Unfolding kinetics studied by steady-state fluorescence measurements

**Figure 4:**
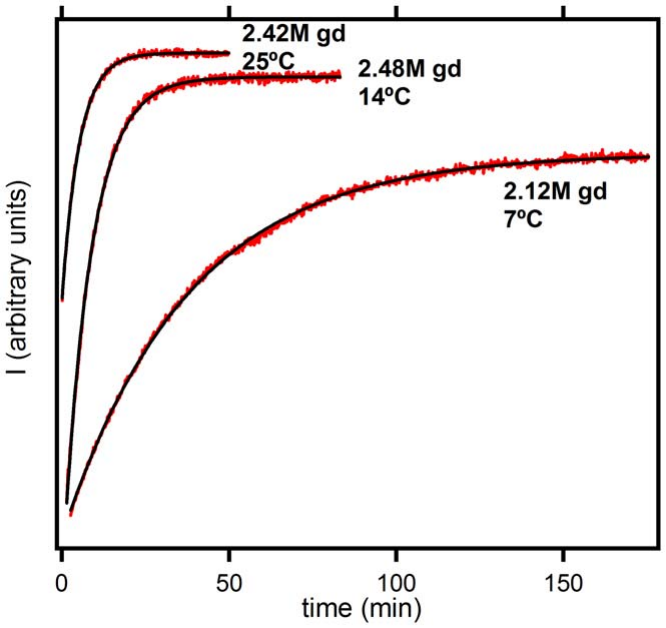
Representative examples of fluorescence intensity versus time profiles for thioredoxin unfolding. The numbers alongside the profiles stand for the temperature and the guanidine concentration used. Experimental data are shown in red and the black line represents the best fit of a single-exponential.

Unfolding kinetics in 50 mM Hepes pH 7 in the presence of different guanidine concentrations were monitored by following the time dependence of the fluorescence emission at 350 nm (excitation at 276 nm) after suitable guanidine concentration jumps (100-fold dilution from zero guanidine concentration). Experiments were carried at different temperatures (7°C, 14°C, 25°C, 30°C and 37°C) and, for each temperature, at several guanidine concentrations. See Figure 4 for representative examples of the experimental fluorescence intensity versus time profiles. Values of the first-order rate constant (k_U_) were calculated from fittings of a single exponential to the experimental fluorescence intensity versus time data.

For each temperature, plots of logarithm of rate constant versus guanidine concentration (C) were linear (see Figure 2b), indicating that the experimentally studied guanidine concentration ranges correspond to the unfolding branches of the chevron plots. The linear dependence can be written as,
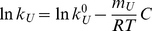
(1)


where m_U_ is the unfolding kinetic m-value, i.e., the minus derivative of the unfolding activation free-energy with respect to the concentration of chemical denaturant. Kinetic m_U_ values are commonly taken as a measure of exposure to solvent of the folding-unfolding transition state as compared with that of the native state. Experimental kinetic m_U_ values for thioredoxin unfolding (derived from the slopes of the plots in Figure 2b) do not significantly change with temperature within the studied range (7–37°C). k_U_^0^ in equation 1 is the linear-extrapolation estimate of the unfolding rate constant for zero guanidine concentration. The values thus extrapolated (see values labeled “0M” on Figure 2c) must be viewed with due caution, as deviations from the linear extrapolation model have been reported at low guanidine concentrations and linked to the screening of charge-charge interactions [49]. Still, the extrapolated values are very useful as a qualitative indication of the time-scale of the unfolding process in the absence of chemical denaturants (see Figure 2c). Furthermore, deviations form the linear-extrapolation model have been detected at low guanidine concentration [49] and do not affect the extrapolation/interpolation of the linear dependencies to 2M guanidine (i.e., the values labeled “2M” in Figure 2c).

The value of lnk_U_ at 2M guanidine increases with temperature in a clearly nonlinear fashion (see Figure 2c) and, indeed, Arrhenius plots (not shown) are clearly non-linear. Within the context of transition-state theory, this may be rationalized in terms of a significant value of the activation heat capacity change, although, a rugged energy landscape (implying super-Arrhenius dependence) could also contribute to the observed nonlinearity [50]–[52]. For the purpose of the computational simulations of denaturation profiles (see further below) we have bypassed the issue of the molecular origin of the non-linear temperature dependence and used instead the following phenomenological equation to describe the k_U_(T) dependence:
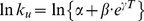
(2)


where α, β and γ are fitting parameters. Fits of equation 2 to the experimental lnk_U_ values are shown in Figure 2c.

### Computational simulation of heat-cold denaturation profiles including the effect of folding-unfolding kinetics

The simulation procedure we have used is essentially that described by Plaza del Pino et al. [31]. Briefly, a temperature-scanning experiment can be seen as a series of very small, equally-spaced temperature jumps. For a two-macrostate (native-unfolded) model, the relaxation kinetics after the jump from T_i_ to T_i+1_ is given by,
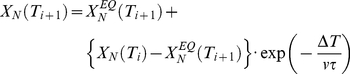
(3)


where X_N_ is the fraction of native protein, v is the scanning rate, ΔT = T_i+1_−T_i_ ( = T_i+2_−T_i+1_ = T_i+3_−T_i+2_ = …) and τ is the folding-unfolding relaxation time,
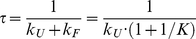
(4)


where we have used that the unfolding equilibrium constant equals the ratio of the unfolding to the folding rate constants (K = k_U_/k_F_). The fraction of native protein at equilibrium in equation 4 is also related to K,
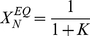
(5)


Accordingly, X_N_ versus temperature profiles can be computed from the repeated application of equation 3, provided that the temperature dependencies of K and k_U_ are known or assumed. These profiles can in turn be transformed into simulated DSC thermograms by using,
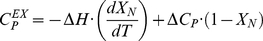
(6)


where C_P_^EX^ is an excess heat capacity defined with respect to the native heat capacity level, and ΔC_P_ and ΔH are, respectively, the unfolding changes in heat capacity and enthalpy.

Application of equations 3–6 to the simulation of denaturation profiles requires that the temperature dependencies of the rate constant k_U_, the equilibrium constant K, the unfolding enthalpy (ΔH) and heat capacity (ΔC_P_) are known (or reasonably estimated) as a function of temperature. The temperature dependence of the unfolding rate constant is known from experiment and adequately represented (Figure 2c) by equation 2. The temperature dependencies of K, ΔH and ΔC_P_ can be described on the basis of well-known, straightforward thermodynamics:
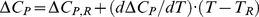
(7)

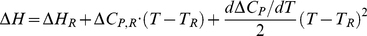
(8)

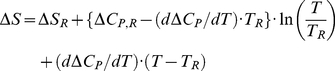
(9)

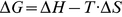
(10)

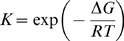
(11)


In the above equations ΔS and ΔG are, respectively, the unfolding changes in entropy and free energy (the latter being immediately related with the value of the equilibrium constant through equation 11), ΔH_R_, ΔS_R_ and ΔC_P,R_ are the unfolding changes in enthalpy, entropy and heat capacity at a given reference temperature (T_R_) and, finally, we take into account the possibility that the unfolding heat capacity change depends on temperature according to a constant slope dΔC_P_/dT.

Simulations were started at a temperature of 2°C with the equilibrium distribution of protein states at that temperature. This is intended to mimic the experimental protocol, which involved a 180 min equilibration at 2°C before the start of the upward temperature-scan. In all the computer simulations shown (Figure 3c) a very small temperature interval (ΔT in equation 3) of 0.04 degrees was used. Nevertheless, we checked that using ten times-larger intervals led to the same results.

The energetic parameters used for the simulations shown in Figure 3c are:

heat denaturation temperature = 49°C,

unfolding enthalpy change at 25°C = −51.6 kJ/mol (note that 25°C is below the enthalpy inversion temperature and, consequently, ΔH<0 at 25°C),

unfolding heat capacity change at 25°C = 7.2 kJ·K^−1^·mol^−1^,

dΔC_P_/dT = 0.1 kJ·K^−2^·mol^−1^.

These values were arrived at as follows. Initial simulations were carried using the literature estimates of the unfolding heat capacity change, the heat-denaturation temperature and the unfolding enthalpy [53], [54]. It was soon realized, however, that simulations using a temperature-independent unfolding heat capacity (i.e. dΔC_P_/dT = 0 in equations 7–9) could not reproduce the experimental denaturation profiles and that a positive dΔC_P_/dT value was required to bring cold denaturation within the studied temperature range. It is interesting that dΔC_P_/dT>0 implies that the ΔC_P_ value at cold-denaturation temperatures is smaller than the value at heat-denaturation temperatures, and, therefore, may be interpreted as suggesting hydrophobic burial in the cold-denatured state. Finally, the energetic parameters were slightly modified (i.e., fine-tuned) to achieve a qualitative agreement of the predicted denaturation profiles with the experimental ones. Note, however, that the description of the folding-unfolding kinetics (based upon the experimental unfolding rate date of figure 2c, together with equations 2, 4 and 5) was not modified or fine-tuned in any way.
